# Eco-evolutionary dynamics sustain a potent yet rare antibiotic gene cluster in *Streptomyces*

**DOI:** 10.1093/ismejo/wrag060

**Published:** 2026-03-18

**Authors:** Jiao Wang, Ning Liu, Minghao Liu, Ying Huang

**Affiliations:** State Key Laboratory of Microbial Diversity and Innovative Utilization, Institute of Microbiology, Chinese Academy of Sciences, Beijing 100101, China; College of Life Sciences, University of Chinese Academy of Sciences, Beijing 100049, China; State Key Laboratory of Microbial Diversity and Innovative Utilization, Institute of Microbiology, Chinese Academy of Sciences, Beijing 100101, China; State Key Laboratory of Microbial Diversity and Innovative Utilization, Institute of Microbiology, Chinese Academy of Sciences, Beijing 100101, China; State Key Laboratory of Microbial Diversity and Innovative Utilization, Institute of Microbiology, Chinese Academy of Sciences, Beijing 100101, China; College of Life Sciences, University of Chinese Academy of Sciences, Beijing 100049, China

**Keywords:** *Streptomyces*, *Streptomyces albidoflavus*, eco-evolution, microbial secondary metabolite, biosynthetic gene cluster, horizontal gene transfer, kosinostatin, antibiotic, microcosm experiments, fitness

## Abstract

Microbial secondary metabolites have been recognized and utilized for nearly a century. Nevertheless, the eco-evolutionary mechanisms governing their distribution among microorganisms remain largely unresolved. In this study, we examined intraspecific interactions within *Streptomyces albidoflavus* and identified a strain exhibiting potent antagonistic activity against conspecifics. This “killer” phenotype was attributed to the production of kosinostatin, a hybrid aromatic polyketide antibiotic. Evolutionary genomic analyses provided strong evidence that the kosinostatin biosynthetic gene cluster was horizontally acquired in *S. albidoflavus* over a relatively short evolutionary timescale, a finding consistent with its sparse distribution within this species, across the genus *Streptomyces*, and even throughout the phylum *Actinomycetota*. Using microcosm assays, we demonstrated that the kosinostatin producer outcompeted sensitive conspecifics in liquid culture but not in soil, indicating that environmental context plays a key role in altering the fitness benefits of this cluster. Moreover, the competitive advantage was observed only in the presence of sensitive strains, revealing a trade-off between fitness benefits and metabolic costs. These results highlight the role of context-dependent selection in shaping the evolutionary persistence of the kosinostatin cluster. The current distribution pattern of this cluster in *S. albidoflavus* likely results from a dynamic interplay of intraspecific horizontal gene transfer, vertical inheritance, and recurrent gene loss. Overall, our findings establish an eco-evolutionary framework that explains the rarity of a potent antibiotic gene cluster in *Streptomyces*, illustrating how environmental constraints, fitness trade-offs, and gene flux collectively orchestrate the biosynthetic architecture of *Streptomyces* species.

## Introduction

Microbial natural products, often referred to as microbial secondary metabolites (SMs), are the main source of antibiotics that have widespread applications in medicine and agriculture [1]. SMs are also widely expected to play a vital role in mediating communication and interactions within and between species in the microbial world [1–3]. Genes responsible for SM biosynthesis typically co-localize into gene clusters, facilitating their horizontal transfer and adaptive evolution [4, 5]. Biosynthetic gene clusters (BGCs) could be inherited vertically, indicating that SMs can serve as functional traits used to define species [6]. In contrast, horizontal gene transfer (HGT) of BGCs is common in natural bacterial groups [7, 8]. In addition, gene loss, duplication, and divergence have also been proved important in the evolution of BGCs [6, 9, 10]. Collectively, these processes structure the distribution pattern of BGCs in microbial populations or species.

SMs are generally retained when they confer a selective advantage to their producers in specific environments [11, 12]. Thus, their distribution pattern could be linked to different evolutionary processes and used to inform ecological interactions they involved. In the postgenomic era, the development of genome sequencing technologies and bioinformatics tools has enabled us to reveal the microbial BGC diversity and distribution patterns at the levels from genus down to strains [6, 13]. However, the eco-evolutionary forces driving the diversity of microbial secondary metabolome are poorly characterized, with only a few systems understood to date. An ecological explanation for the population-specific distribution pattern of aflatoxin in the fungus *Aspergillus flavus* has been proposed [14], and several studies have inferred the evolutionary history of BGC patterns in bacteria [6, 11, 15, 16]. These works set important precedents for linking BGC diversity to its underlying eco-evolutionary drivers.

*Streptomyces* bacteria, typified by their complex lifestyle and important roles in global nutrient cycling, are the most prolific producers of useful antibiotics [17, 18]. The ever-increasing genomic data have revealed the presence of a vast potential BGC reservoir in *Streptomyces* [19]. Despite being termed “secondary” metabolites, high-frequency or core SMs have been proved to be essential for *Streptomyces* competition and thriving [12, 20]; and population-specific SMs likely underpin environment adaptation [21]. Nevertheless, although comparative genomics has revealed extensive intraspecific BGC diversity in *Streptomyces* [22, 23], we are still at the beginning of understanding the ecological roles of low-frequency SMs due to their high diversity, sparse distribution, and inherent challenges in detection. The eco-evolutionary mechanisms sustaining such high intraspecific BGC diversity in this genus thus remain unclear. Addressing these questions will likely provide new insights into the persistent conundrum of high rediscovery rates in natural product discovery. This requires linking genetic variation to phenotypic heterogeneity—a particular challenge for BGCs given their frequent cryptic or silent nature [24, 25]. Given that SMs are known to mediate ecological interactions among strains [1–3], quantifying interaction patterns could provide clues about how intraspecific BGC variation affects species fitness. Thus, integrating intraspecific BGC distribution analyses with interaction assays may enable us to generate testable hypotheses about evolutionary dynamics shaping BGC diversity and gain mechanistic insights into eco-evolutionary feedback.

*Streptomyces albidoflavus*, one of the most geographically ubiquitous *Streptomyces* species, serves as a model system for studying bioactive natural products and adaptive evolution [23, 26, 27]. A previous study characterized its BGC pattern using seven strains [23], highlighting strain-level SM diversity within this species. Currently, *S. albidoflavus* hosts the largest collection of publicly available genomes among all described *Streptomyces* species. Here, we expanded this genomic resource to 48 strains to resolve its species-level BGC pattern. Through systematic pairwise interaction profiling across 25 *S. albidoflavus* strains, we identified a killer strain capable of suppressing conspecific growth. Subsequent metabolite analysis and genetic validation revealed kosinostatin, a low-frequency bioactive SM, as the driver of intraspecific inhibition. Evolutionary and comparative genomics analyses further indicated HGT as the origin of the kosinostatin BGC in *S. albidoflavus*. Finally, by integrating microcosm experiments with population genomic data, we propose an eco-evolutionary explanation to the apparent contradiction of the persistent rarity of this potent SM in *Streptomyces*.

## Materials and methods

### Pairwise head-to-head competition assays

To investigate intraspecific interactions within *S. albidoflavus*, we utilized a collection of 25 strains previously described and sequenced in our earlier work [26, 27]. These strains were first cultured on GYM agar (glucose 4.0 g, yeast extract 4.0 g, malt extract 10.0 g, CaCO_3_ 2.0 g, and agar 15.0 g in 1 liter dd-H_2_O; pH 7.0) at 28°C for 7 days. Spores were then harvested and resuspended in dd-H_2_O to a final concentration of ~1 × 10^7^ spores per ml. For competition assays, 3 μl of spore suspensions from each of two strains were simultaneously spotted onto GYM agar plates, with inoculation points spaced 0.5 cm apart. Control plates were inoculated with the same strain spotted against itself. All plates were incubated at 28°C for 7–20 days, and all assays were performed in triplicate. Interactions were classified as neutral if no change in colony size was observed for both strains compared to the controls, and as inhibitory if a decrease in colony size occurred. To differentiate interference from exploitation competition, agar diffusion assays were performed as previously described [28], with modifications detailed in Supplementary Methods.

### Bioassay-guided fractionation and elucidation of the bioactive compound

The bioactive compound responsible for the inhibition was identified from strain FXJ6.189. Following ethanol extraction of the fermented agar, the crude extract was fractionated by HPLC. Bioactive fractions were identified using a well-diffusion assay, and the active constituent was ultimately elucidated as kosinostatin through high-resolution mass spectrometry (HR-ESI-MS). Full experimental details are provided in Supplementary Methods.

### Construction of gene disruption mutants

To confirm the biosynthetic origin of kosinostatin, a disruption mutant of the key gene *ksnC3* [29] was constructed in strain FXJ6.189. Using Gibson assembly, a knockout vector was created and introduced into FXJ6.189 via conjugation from **Escherichia* coli* ET12567/pUZ8002. Mutants (FXJ6.189Δ*ksnC3*) were selected and verified by PCR and sequencing (see Supplementary Methods for details). Independent mutant clones were assessed for kosinostatin production and antimicrobial activity.

### Microcosm experiments

To assess the effects of kosinostatin production on producer and population fitness, we performed co-culture experiments in liquid and soil microcosms using producer strain (FXJ6.189), its isogenic non-producing mutant (FXJ6.189Δ*ksnC3*), and a kosinostatin-sensitive strain (CR15, randomly selected). Due to the lack of easily discernible morphological features or a specific genetic maker for strain differentiation, we chromosomally integrated genes encoding fluorescent proteins sfGFP (green), mCherry (red), and mOrange (orange) into the producer, sensitive, and non-producer strains, respectively. These fluorescent reporter genes were cloned under the constitutive *hrdB* promoter into the integrating plasmid pSET152 [30], which was then introduced into streptomycetes via conjugation. Control experiments with systematically rotated fluorescent tags among strains confirmed that the tag choice had no measurable effect on strain fitness (data not shown). All strains and plasmids used in this study are summarized in Table S5, and primers are listed in Table S6.

To test whether the *ksn* cluster confers a direct competitive benefit under different conditions, the producer strain was co-cultured with the sensitive strain at an initial ratio of 1:9 (producer:sensitive) in liquid and 1:1 in soil microcosms. Its isogenic non-producer served as a control under identical conditions. To evaluate the intrinsic metabolic cost of kosinostatin production, the producer and non-producer were co-cultured at an initial ratio of 1:1 in liquid microcosms. To assess if the benefit requires a sensitive target, a three-strain community (producer:non-producer:sensitive strain at 1:1:18) was established in parallel liquid microcosms. All experiments were performed with three biological replicates.

**Liquid microcosm procedure**—Liquid co-cultures were established in 10 ml of SPA medium (starch 10.0 g, proline 0.4 g, asparagine 0.4 g, KNO_3_ 2.0 g, NaCl 2.0 g, K_2_HPO_4_ 2.0 g, MgSO_4_ 0.05 g, and FeSO_4_ 0.01 g in 1 liter dd-H_2_O; pH 7.0) [31] in a 50-ml plastic screw-cap tube. Pre-germinated spores of each strain were adjusted to a concentration of 10^4^ spores/μl in sterile water. Co-cultures were inoculated with 1 ml of spore mixture at the defined ratios, incubated at 28°C with shaking (200 rpm), and serially passaged (1:10 dilution into fresh medium) every 3 days for a total of 24 days (8 transfers). Before each transfer, the tubes were shaken violently to homogenize the cultures. After each transfer, DNA was extracted from the remaining culture for subsequent amplicon sequencing to quantify strain frequencies and monitor population dynamics. In parallel, an ethanol extract of the culture was prepared for bioactivity assessment by well-diffusion assay.

**Soil microcosm procedure—**The soil used in the co-culture system was collected from the topsoil (5–20 cm depth) in northern Jiangxi Province, China. After air-drying, the soil was sieved through a 0.15-mm sieve and sterilized by autoclaving twice at 121°C and 100 kPa for 20 min with a 24 h interval. It was then dried in an oven for several days until a constant weight was achieved. Each soil microcosm consisted of 7 g soil in a 50-ml glass beaker. Spore suspensions were mixed to achieve a 1:1 ratio and a final concentration of 10^4^ spores/μl SPA medium. A volume of 1050 μl of suspensions were added to the beaker to achieve 15% (w/w) soil moisture. Beakers were sealed and incubated at 28°C for 28 days. Triplicate microcosms per setup were destructively harvested every 7 days for DNA extraction and amplicon sequencing; strain growth was quantified by dilution plating and colony-forming unit enumeration.

### DNA extraction and amplicon sequencing

DNAs from liquid and soil microcosms were extracted using the Solarbio Bacterial DNA Extraction kit (catalogue no. D1600) and the Qiagen DNeasy PowerSoil kit (catalogue no. 12888), respectively. To distinguish the three engineered *S. albidoflavus* strains, which share nearly identical genetic backgrounds, a 700-bp fragment within the integrated fluorescent protein genes was selected for primer design (FP-For/FP-Rev). A set of 8-bp barcode oligonucleotides were incorporated into the forward primers. PCR amplification was performed using the extracted DNAs as templates. All PCR products were sequenced on a MiSeq System (Illumina) by GENEWIZ Biological Technology Co., Ltd (Suzhou, China; https://www.genewiz.com.cn). Reads containing barcodes exactly matching the fluorescent protein genes were extracted, and the relative abundance of each strain was quantified using a custom Python script. This approach yielded over 10 000 high-quality reads per sample.

### Identification of putative kosinostatin BGCs in the phylum *Actinomycetota*

A total of 34 389 *Actinomycetota* genomes (accessed in December 2021) were retrieved from the National Center for Biotechnology Information (NCBI) using the ncbi-genome-download Python script (https://github.com/kblin/ncbi-genome-download/). Protein-coding sequences were predicted from all genomes with Prodigal v2.6.3 [32] under default parameters. The resulting amino acid sequences were used to create a local BLAST database via the makeblastdb utility of NCBI BLAST+ 2.6.0. A set of 33 structural genes conserved between the *kst* and *ksn* clusters (Table S3) was used as a query for a blastp search with the following parameters: identity >30%, query coverage >50%, and E-value <1e-5. Putative kosinostatin BGCs were identified using a custom Python script to select genomes containing at least 27 (80%) of the query genes. A threshold of 80% was chosen as it reproduced the results of the more stringent 90% threshold while avoiding the false positives (partial BGCs) observed at 70%. These candidate genomes were further analyzed with antiSMASH v.5 [33], and the results were manually curated to confirm the presence of a complete kosinostatin BGC.

### CAI calculation

Genes (excluding those of the *ksn* cluster) in *S. spiroverticillatus* JCM 4609^T^ and five *ksn*-harboring *S. albidoflavus* strains were categorized as either annotated or hypothetical based on Prokka v1.14.6 [34] annotations. CAI was calculated for annotated genes, hypothetical genes, and *ksn* genes in each genome using CodonW software (John Peden, https://sourceforge.net/projects/codonw/), with the full set of annotated genes serving as the reference. Differences in CAI among groups were assessed by one-way analysis of variance in R v4.2.0 [35], followed by Tukey’s honest significant difference test for post-hoc comparisons.

### Phylogenetic analyses

A total of 67 strains classified as *S. albidoflavus* in the EzBioCloud database [36] were initially included (Table S7). After quality control (excluding strains with more than 500 contigs) and dereplication, 51 strains were retained for phylogenetic reconstruction. Among these, 48 strains formed a core group, sharing average nucleotide identity (ANI) values >98.6% (mean = 98.9%), whereas the remaining three (PVA 94–07, GBA 94–10, and ADI 96–15) showed ANI < 96.4% with the core. Following our previous study [26], these three divergent strains were designated as the outgroup. Orthogroups were identified using GET_HOMOLOGUES [37] with OrthoMCL algorithm. Single-copy orthologous genes conserved across all 51 genomes were aligned at the amino acid level using MAFFT v7.407 [38] under the L-INS-i strategy, and then converted to nucleotide alignment through PAL2NAL [39]. The alignments were curated with Gblocks [40] (−t = c) and concatenated using a custom script. Phylogenetic inference was performed on the final alignment using FastTree v2.1.10 [41] under the GTR model, and the resulting tree was visualized using iTOL [42]. Similarly, a phylogenetic tree based on *ksn* gene sequences was constructed using the same method, with the *ksn* sequence from *S. spiroverticillatus* JCM 4609^T^ included as an outgroup.

### Identification of BGC pattern in *S. albidoflavus*

**Figure 1:**
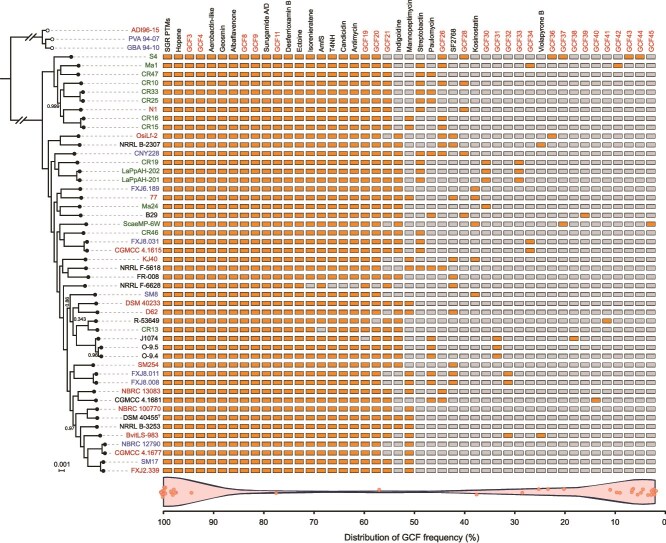
Distribution pattern of BGCs across *Streptomyces albidoflavus* strains. BGCs were classified into gene cluster families (GCFs), which are displayed above the heatmap. Known GCFs are in black, and unknown GCFs in red. Cells within the heatmap indicate the presence (orange) and absence (grey) of each GCF. The tree on the left is a maximum-likelihood phylogeny generated from concatenated 3241 single-copy core genes of 48 *S. albidoflavus* strains (solid dots) and three closely related outgroup strains (hollow dots). Bootstrap values less than 100% are shown at the nodes. Branch lengths are scaled in substitutions per site, as indicated by the number above the scale bar. Strain numbers are colored according to their isolation sources: soil (red), aquatic (blue), insect-associated (green), and unknown (black). The scale bar represents nucleotide substitutions per site. The horizontal violin plot at the bottom illustrates the frequency distribution of GCFs across strains, with the probability density shown by the shape and individual values overlaid as jittered points.

Secondary metabolite BGCs in *S. albidoflavus* were predicted using antiSMASH v.5.0 [33]. These BGCs were subsequently categorized into GCFs through sequence similarity network analysis using BiG-SCAPE [43] with default parameters. The core backbone genes within each GCF were validated using BLASTP [44]. Fragmented BGCs arising from incomplete genome assemblies were resolved through network analysis complemented by BLASTP verification. After deduplication, a strain-resolved profile of BGC distribution across *S. albidoflavus* was generated (Fig. 1). Furthermore, the contigs of all strains were reordered using RagTag [45] with the *S. albidoflavus* KJ40 genome as the reference. Subsequently, the genomic coordinates of all BGCs were transferred and unified onto this reference genome coordinate system according to the NUCmer [46] alignment results (Fig. 1).

### Ancestral state reconstruction of the kosinostatin BGC

The ancestral states of the kosinostatin BGC were reconstructed using COUNT under the phylogenetic birth-and-death model [47]. The analysis took two inputs: the *S. albidoflavus* species tree (core-genome phylogeny) and a presence/absence matrix of the kosinostatin BGC across all strains. Using a maximum-likelihood framework, the model inferred ancestral presence/absence at each node and estimated the probabilities of gain and loss events along each branch.

## Results

### BGC pattern in *S. albidoflavus*

A total of 1120 BGCs from 48 *S. albidoflavus* genomes (contig number < 500) was identified by using antiSMASH 5.0 [33] and manual curation of characterized clusters. Network analysis of sequence similarity grouped these BGCs into 45 Gene Cluster Families (GCFs), including 20 high-frequency GCFs (GCF1–GCF20) shared among more than 80% individuals, 18 low-frequency GCFs (GCF28–GCF45) present in less than 20% individuals, and 7 medium-frequency GCFs (GCF21-GCF27) (Fig. 1). This result indicated a high strain-level diversity of BGCs in *S. albidoflavus*. To investigate the forces shaping the patchy distribution of these low-frequency BGCs, we examined the correlation between their presence/absence patterns and both phylogenetic and geographic distances using Mantel tests (see Supplementary Methods). The results revealed only a weak, albeit significant, correlation with geographic distance (*r* = 0.10, *P* value *=* 0.05) and no significant correlation with phylogenetic distance (*r* = 0.07, *P* value = 0.08).

Among the 20 high-frequency GCFs, 12 encode known metabolites, which have been reported as core secondary metabolome of *S. albidoflavus* in a previous study [23]. Beyond their antibiotic functions (mainly antifungal), the core SMs also significantly contributes to resource acquisition (i.e. siderophores), stress resistance (i.e. ectoine and tetrahydroxynaphthalene), and life cycle regulation (i.e. AmfS and geosmins), highlighting their essential biological and ecological functions (Table S1). In contrast to the high-frequency GCFs, however, only two (GCF29 and GCF35) of the 18 low-frequency GCFs show considerable homology to BGCs encoding known products, the antibiotics kosinostatin and violapyrone B. These results indicate that little is known about the attractive low-frequency SM repertoire in *S. albidoflavus*, despite extensive research on secondary metabolism of this species. Genomic mapping revealed that these GCFs, including low-frequency ones, are scattered across the entire genome (Figs. S1 and S2). This pattern indicates that gene cluster gain and loss in *Streptomyces* is not confined to the active chromosomal arms [48, 49]. Additionally, two functionally similar GCFs (both involved in metal ion transport) are located at the same genomic locus (Fig. S3), suggesting a possible case of functional replacement.

### Discovery of a killer in *S. albidoflavus* and characterization of the bioactive compounds

To explore whether SMs contribute to fitness difference among conspecific strains, we conducted pairwise interaction across 25 strains of *S. albidoflavus* using a head-to-head competition assay (Fig. S4). Most interacting pairs (57.3%) showed neutral effects (Table S2 and Fig. S5), that is, the two strains did not affect the growth of each other in the interaction. The remaining pairs exhibited unidirectional inhibition, where one strain suppressed the other’s growth to varying degrees (Table S2 and Fig. S5). Among these, a killer strain, FXJ6.189, exhibited inhibitory activity against its conspecific strains across all pairwise interactions (Figs. S4 and S5). Further agar diffusion assays using cell-free agar blocks from the FXJ6.189 culture demonstrated that the inhibitory activity was mediated by diffusible metabolites produced by this strain (Fig. S6A).

To identify the inhibitory metabolites in culture extracts of strain FXJ6.189, we used a bioassay-guided fractionation approach (Fig. S6B and C) followed by structural elucidation employing HR-ESI-MS. This led to targeting two bioactive compounds characterized by the same ultraviolet (UV) absorbance at 231, 258, 290 (sh), and 433 nm and molecular mass corresponding to [M + H]^+^ = 617.213 (Fig. S7). After database retrieval from the Dictionary of Natural Products online (https://dnp.chemnetbase.com/), we identified the compounds as kosinostatin and its naturally occurring isomer isoquinocycline B [50, 51] (Fig. S7). Kosinostatin is one of the most complex hybrid aromatic polyketide natural products and shows significant antimicrobial activity against Gram-positive bacteria [52]. The BGC of kosinostatin (*kst*) has been identified and characterized in *Micromonospora* sp. TP-A0468 [29].

### Identification and evidence for horizontal acquisition of the kosinostatin gene cluster in *S. albidoflavus*

**Figure 2:**
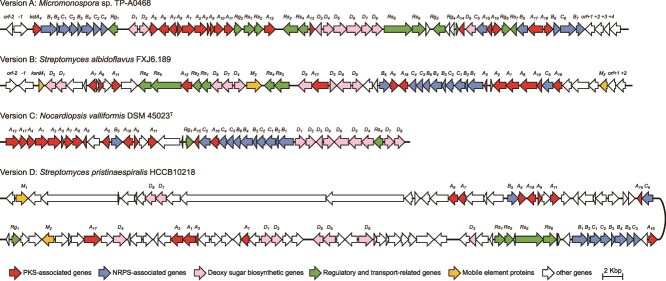
Organization and structural diversity of kosinostatin BGCs across *Actinomycetota*. Genes not identified in the known kosinostatin producer *Micromonospora* sp. TP-A0468 are shown in white. Versions A and B were experimentally validated, whereas versions C and D were predicted via bioinformatics analysis. Owing to its length, the gene cluster in version D is split across two rows; a black curve connects the segments to denote their contiguous genomic arrangement.

AntiSMASH identified a putative gene cluster for kosinostatin in strain FXJ6.189, named *ksn*. Further BLAST analysis revealed that most genes in this cluster share high similarity with their counterparts in cluster *kst*, but with a rearranged gene order (Fig. 2 and Table S3). To determine if *ksn* is responsible for kosinostatin biosynthesis in strain FXJ6.189, we disrupted the structural gene *ksnC3* (encoding a putative phosphoribosylanthranilate isomerase) via replacement with a kanamycin-resistance gene *neo*. The mutant strain FXJ6.189Δ*ksnC3* abolished production of both kosinostatin and isoquinocycline B and lost inhibitory activity, as confirmed by HPLC and HR-ESI-MS analyses as well as antimicrobial assay (Figs. S6D and S7). These results establish that the *ksn* cluster governs kosinostatin production and confers antagonism in *S. albidoflavus* FXJ6.189.

**Figure 3:**
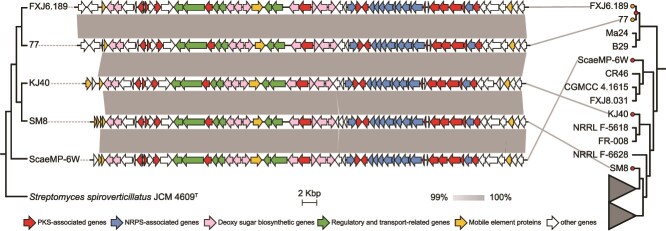
Genomic organization, distribution, and evolution of the *ksn* gene cluster in *S. albidoflavus*. Gene organization of *ksn* clusters is depicted in the center. Grey bars represent BLASTN identity, with a grey gradient representing the range from 99% to 100%. The left panel shows a phylogenetic tree reconstructed from *ksn* gene sequences. The right panel displays a phylogenomic tree (reproduced from Fig. 1), illustrating the phylogenetic positions of the five *ksn*-carrying strains within the broader genomic context of *S. albidoflavus*. Some clades have been collapsed for clarity. Ancestral state reconstruction of the *ksn* gene cluster is mapped onto the phylogenomic tree; HGT and vertical transmission events are marked with red and orange dots, respectively (posterior probability = 1.0 for all inferred events).

The BGC pattern of *S. albidoflavus* showed that the *ksn* cluster was absent in all other *S. albidoflavus* strains used in the head-to-head competition assay. This cluster was a low-frequency BGC found in only five *S. albidoflavus* strains, which were sporadically distributed in the phylogenomic tree of *S. albidoflavus* (Fig. 3). Synteny analysis indicated that all five *ksn* clusters reside at a shared locus within the chromosomal terminal regions (Fig. S8). Further bioinformatics analysis showed that these clusters were all flanked by transposase-coding genes on both sides. Together, these observations suggest that *ksn* in *S. albidoflavus* was acquired by HGT, which is supported by ancestral reconstruction analysis of *ksn* (Fig. 3)*.* Codon adaptation index (CAI) analysis showed a significant difference (*P* value <0.001) between *ksn* genes and other annotated genes in the genomes of *S. albidoflavus* (Fig. S9), indicating that codon usage of the horizontally acquired *ksn* cluster has not been normalized to the recipient genomes.

### Diversity and evolutionary dynamics of kosinostatin BGCs within *Streptomyces* and across the phylum *Actinomycetota*

Screening of 34 389 publicly available *Actinomycetota* genomes (comprising 2526 from 586 *Streptomyces* species) revealed the extreme rarity of the kosinostatin BGC. Only 18 putative carriers were identified, 13 of which were from *Streptomyces*, representing a mere 0.5% of the *Streptomyces* genomes surveyed. The positive genomes derive from 15 dereplicated strains of three genera: *Micromonospora* (2 strains), *Nocardiopsis* (3), and *Streptomyces* (10). These strains were isolated from diverse habitats including various soils (e.g. rhizosphere, saline, desert), sponges, seawater, insect, and plant leaves (Table S4). Comparative analysis of gene content and order revealed four versions (A-D) of the kosinostatin BGCs (Fig. 2). Version A was derived from *Micromonospora*, and version B was found in *S. albidoflavus* and *Streptomyces spiroverticillatus*. Both versions A and B have been experimentally linked to the production of kosinostatin. Version C, which was exclusive to *Nocardiopsis*, exhibits a more modular genetic organization comprising three subclusters and fewer regulatory genes, consistent with an earlier evolutionary origin. Version D was identified in *Streptomyces pristinaespiralis*, where it is integrated into a ~ 210 kb supercluster that biosynthesizes at least three different SMs [53].

Although *S. spiroverticillatus* JCM 4609^T^ is phylogenetically distant from *S. albidoflavus* (with an ANI of only 85.5%), their kosinostatin BGCs are highly similar (Fig. S10), sharing nearly identical gene order and at least 90% sequence similarity. The presence of multiple transposase-coding genes within and flanking the *ksn* cluster of *S. albidoflavus*, in contrast to their complete absence from the counterpart in *S. spiroverticillatus*, suggests that the cluster was acquired by *S. albidoflavus* via HGT from *S. spiroverticillatus*. This inference is further supported by CAI analysis (Fig. S9), which revealed significant codon usage bias between the *ksn* genes and other annotated genes in *S. albidoflavus* but not in *S. spiroverticillatus*.

### Fitness benefits and costs of kosinostatin production in *S. albidoflavus*

To characterize how the horizontally acquired *ksn* cluster shapes fitness and population dynamics in *S. albidoflavus*, we employed competitive experiments. The kosinostatin producer was co-cultured with isogenic non-producer and/or sensitive strains in liquid and soil microcosms, allowing dynamic monitoring of their interactions.

**Figure 4:**
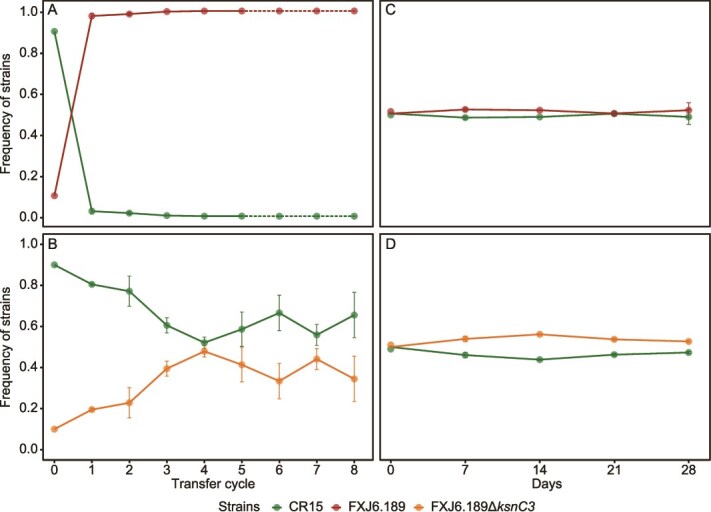
Evolutionary dynamics of two-strain competition in liquid and soil microcosms. (A, B) Dynamics between the kosinostatin-producing strain FXJ6.189 (A) or the non-producing mutant FXJ6.189Δ*ksnC3* (B) and the sensitive strain CR15 (initial ratio 9:1) in liquid microcosms. (C, D) Corresponding dynamics in soil microcosms between FXJ6.189 (C) or FXJ6.189Δ*ksnC3* (D) and CR15 (initial ratio 1:1). Data points represent mean strain frequencies from three biological replicates, with error bars indicating the standard error of the mean. Data collection for (A) was terminated after generation five following the extinction of strain CR15. The dashed line beyond this point denotes a value of zero for the extinct strain and the resulting monoculture of the winner.

In liquid co-cultures initiated at a 1:9 ratio (producer:sensitive or non-producer:sensitive), the sensitive strain was rapidly eliminated by the producer before the first passage, whereas it reached a stable equilibrium with the isogenic non-producer after the fourth passage (Fig. 4A and 4B). This result indicates that kosinostatin production confers a selective advantage on the producer strain, leading to a loss of strain diversity in this synthetic community. In two-strain soil microcosms, however, different dynamics were observed. Given that streptomycetes grow slowly in soil, a 28-day cultivation was conducted and dilution plating was used to confirm the growth of strains (Fig. S11). Throughout the cultivation, the sensitive strain stably coexisted with both the producer and non-producer strains at the initial 1:1 ratio (Fig. 4C and D). This suggests that the competitive advantage conferred by the *ksn* cluster is absent under these soil conditions. The stark contrast between effective elimination in liquid and stable coexistence in soil suggests that the fitness benefit of *ksn* carriage in *S. albidoflavus* is contingent upon environmental conditions.

**Figure 5:**
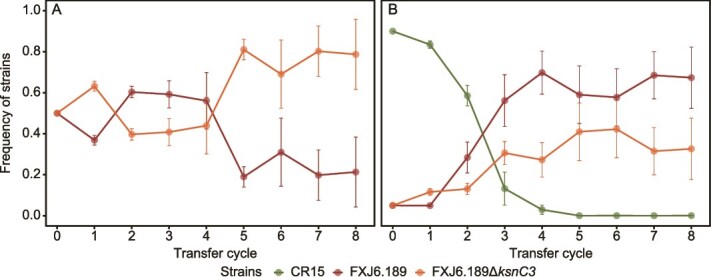
Evolutionary dynamics among the kosinostatin producer (FXJ6.189), resistant (FXJ6.189Δ*ksnC3*), and sensitive (CR15) strains in liquid microcosms. (A) Dynamics between FXJ6.189 and FXJ6.189Δ*ksnC3* (initial ratio 1:1) in the absence of CR15. (B) Three-strain dynamics among FXJ6.189, FXJ6.189Δ*ksnC3*, and CR15 (initial ratio 1:1:18). Data points represent mean strain frequencies from three biological replicates, with error bars indicating the standard error of the mean.

To further investigate selection for kosinostatin production, we compared the relative fitness of the producer and isogenic non-producer in two distinct liquid microcosms. In the absence of a sensitive strain, the non-producer outcompeted the producer by the fifth passage (Fig. 5A), indicating that the metabolic burden of kosinostatin production incurred a fitness cost (Fig. S12). However, this dynamics shifted markedly in the three-strain microcosm containing the sensitive strain. Under these conditions, the producer strain exhibited the highest relative fitness (Fig. 5B). Although the sensitive strain was ultimately eliminated from the population due to antagonistic competition, the extinction occurred at a slower rate compared to the two-strain microcosm in Fig. 4A. These results demonstrate that the fitness benefit of the *ksn* cluster is realized specifically when its product effectively inhibits susceptible competitors. That is, by inhibiting neighboring sensitive cells, the compound allows the producer to exploit more resources.

Collectively, the microcosm experiments described above indicate that the *ksn* cluster provides a fitness benefit to its host, but the specific advantage is defined and modulated by the surrounding abiotic and biotic context.

## Discussion

### Ecological maintenance of kosinostatin BGCs

Although SMs are generally thought to confer adaptive benefits [54], fundamental questions remain regarding how their production shapes strain fitness landscapes, mediates population dynamics, and in turn directs the evolution of their encoding BGCs. In this work, through integrated interaction experiments and bioinformatics analysis, we characterize the puzzling rarity of a BGC (*ksn*) encoding the potent bioactive compound kosinostatin in *S. albidoflavus*. Kosinostatin and its analog isoquinocycline have been known for ~60 years [55, 56]. These aromatic compounds have gained wide attention due to their antimicrobial and antitumor activities [50, 57]. However, the ecological functions of these compounds and the selective forces that maintain these metabolically expensive trait have not yet been investigated, a knowledge gap that extends to the majority of microbial natural products [58]. By experimentally quantifying the fitness effect associated with this horizontally acquired *ksn*, we provide explanations to the rarity of the kosinostatin BGC from the perspective of evolutionary ecology.

Our experiments demonstrate the context-dependent ecological impact of the kosinostatin BGC: it confers a strong competitive advantage in nutrient-rich liquid co-culture, but this advantage is undetectable in mock soil environments (Fig. 4). Concurrently, this BGC imposes a metabolic cost that reduces producer fitness in the absence of competitors (Fig. 5). This context-dependent behavior argues that dynamic ecological interactions play an important role in shaping the evolutionary fate of the kosinostatin BGC. This notion is reinforced by our finding that the presence of the *ksn* cluster is uncorrelated with broad-scale habitats or geographical origins of the strains (Table S4), indicating that stable, habitat-wide selective pressure may be absent or very weak.

BGCs are widely regarded as products of adaptive evolution [11, 54], a view supported by the potent selection we observed under controlled conditions (Fig. 4). However, adaptive processes alone may be insufficient to ensure their long-term persistence when selective pressures are discontinuous. Many BGCs, including *ksn*, are conditionally activated by specific environmental triggers [59]. Consequently, in natural settings, the windows for expression and positive selection may be brief and spatially restricted to microenvironments like the rhizosphere or host tissues. During the extended “silent” periods that likely dominate its existence, the cluster confers no net fitness effect, and its evolutionary fate may therefore largely governed by neutral processes such as genetic drift [10]. In addition, synteny analysis revealed that the *ksn* clusters reside in the chromosomal terminal regions, which can be disadvantageous for both their expression [60] and stable maintenance [61], thereby increasing the likelihood for non-adaptive processes to operate.

In summary, our findings suggest the persistence of this potent trait is not a direct outcome of constant selection but may be dynamically shaped by the strength of ecological interactions and potentially stochastic processes. The spatiotemporally shifting selection landscape prevents the BGC from either fixation or extinction, sustaining it at a low frequency in the population. The *ksn* cluster thus serves as a flexible genomic toolkit that enhances species-level secondary metabolome diversity—a bet-hedging strategy facilitating dispersal and persistence [11].

### Evolution of kosinostatin BGCs

Our study offers insights into the evolution of kosinostatin BGCs in the phylum *Actinomycetota*. Comparative genomic analysis revealed extensive gene rearrangements among distinct BGC versions, pointing to a complex evolutionary history. The ancestral kosinostatin BGC likely featured a compact tripartite architecture comprising polyketide synthase (PKS), nonribosomal peptide synthetase (NRPS), and deoxy sugar biosynthesis modules (Fig. 2). This organization is best preserved in *Nocardiopsis*, a genus known for producing diverse natural products [62], and may reflect the assembly of ancestral modules acquired from various sources. Over time, presumably through gene gain, loss, and rearrangement [63], the modularity of the BGC eroded, a trend particularly evident in the highly derived cluster in *S. pristinaespiralis*. The sporadic distribution of kosinostatin BGCs throughout *Actinomycetota* suggests that considerable cluster diversity has been lost, likely due to rapid turnover erasing most intermediate forms. In addition, the PKS, NRPS, and deoxy sugar biosynthesis modules are far more broadly distributed than the intact cluster, and dozens of partial BGCs retaining two of the three modules remain detectable (Fig. S13). Future studies integrating expanded genomic sampling, module-level comparative analyses, and key enzyme domain-based searches will help reconstruct the full evolutionary trajectory of this GCF.

The conserved synteny and high sequence identity (>99%) among all *ksn* clusters within *S. albidoflavus* indicate a common ancestral acquisition via a single HGT event (Fig. 3). This inference is supported by previous findings that horizontal acquisition and retention of genes between distantly related *Streptomyces* lineages are rare [64]. Moreover, several lines of evidence are consistent with intraspecific HGT of the cluster within this species. The five *ksn*-positive strains were isolated from highly diverse habitats (e.g. soil, insects, sponges) and distant geographical locations (Table S7), arguing against recent clonal expansion as the sole explanation for their distribution. Instead, the cluster locates at the chromosomal terminus, a known recombination hotspot in *Streptomyces* [65], and harbors mobile genetic elements both within and flanking the cluster, implying a history of multiple, independent integration events into this genomic region. Considering that HGT of intact BGCs in *Streptomyces* occurs predominantly within a 10-million-year window [64], the estimated divergence time of ~5.31 million years between *ksn*-positive and *ksn*-negative *S. albidoflavus* strains (confidence interval, 3.41 to 8.26 mya) (Fig. S14) aligns with intraspecific HGT as a plausible dissemination mechanism [58]. Finally, the phylogenetic incongruence between the *ksn* and species trees of *S. albidoflavus* (Fig. 3) further reinforces HGT within the species. Thus, the distribution pattern of *ksn* within *S. albidoflavus* may be best explained by an initial interspecific HGT acquisition, followed by a combination of vertical inheritance, gene loss, and intraspecific HGT. We hypothesize that after an ancestral acquisition, the cluster’s evolution might be governed by three forces: purifying selection when beneficial, neutral loss when not, and intraspecific HGT that spreads it across the population. The interplay of these processes explains the rare distribution of this cluster while continuously restructuring the genetic landscape of the species.

### Intraspecific competition in *Streptomyces*

Given the genome plasticity of *Streptomyces* and transferability of BGCs [5, 65], it is conceivable that a strain could acquire a BGC encoding a lethal antibiotic from a different species. Such an acquisition could then arm the recipient against conspecific competitors. However, in the context of natural evolution, such a scenario is likely to be transient. Current microbial population profiling approaches, which typically rely on single-time-point or short-time-scale sampling, are poorly suited to capture such fleeting competitive dynamics. A previous study demonstrated that *Streptomyces* populations at a spatial microscale often act as social units, wherein no strain has the ability to inhibit its conspecifics [65]. A similar population organization has also been described in *Vibrio* [66]. In contrast, antagonistic interactions among closely related strains have been well documented in *E*.* coli* [67], which are able to inhibit conspecific relatives by producing bacteriocins [68, 69]. Here, we present an example of intraspecific competition in *Streptomyces*. Despite that the strains we used are not sympatric, they have the possibility to encounter each other in natural settings, given the global distribution of *Streptomyces* and their capacity to produce abundant, readily dispersed spores. Our microcosm experiments demonstrate a potential ecological outcome of such encounters. Under conditions permissive for kosinostatin production and accumulation, the producer strain may outcompete conspecific rivals, potentially leading to a genome-wide selective sweep within the local population. This would purge population diversity under the selective pressure [54, 70], ultimately eliminating conspecifics at a spatial microscale. Although such transient interactions are ecologically significant in shaping population dynamics, they often evade detection due to the limitations of current ecological and genomic sampling methods.

### Limitations and future perspectives

This work is subject to several limitations. First, functional validation of the kosinostatin BGC was performed on a single strain (FXJ6.189). Although the *ksn* cluster is highly conserved in all five carrier strains of *S. albidoflavus* (Figs. 3 and S8), we cannot exclude the possibility of strain-level variation in regulation or expression. Future work should test whether the fitness trade-offs observed here are consistent across different genetic backgrounds. Second, the mechanistic basis of the context-dependent fitness remains incompletely understood. The specific environmental cues (e.g. particular nutrients, quorum-sensing signals) that trigger or silence the BGC expression, along with the corresponding genetic regulatory pathways, await further clarification. In addition, our study relies on simplified laboratory microcosms and short-term data, which cannot capture the complexity of natural environments and long-term evolution. Thus, the fitness trade-offs and evolutionary dynamics we propose call for future validation in more realistic ecological contexts over extended timescales.

Despite the caveats discussed above, our study provides a quantitative, species-level analysis of the eco-evolutionary dynamics of a horizontally acquired potent BGC. Although HGT of BGCs is well-documented, its fitness consequences remain poorly quantified. By focusing on the kosinostatin BGC in *S. albidoflavus*, we develop an eco-evolutionary framework to explain why such potent BGCs remain rare. More broadly, we exemplify a direct link between the bioactivity of a SM and the evolutionary trajectory of its BGC, which may extend to other SMs. Our findings underscore the importance of population-level studies on SMBGCs to unravel their evolutionary mechanisms and ecological roles.

## Supplementary Material

Supplementary_material_wrag060

## Data Availability

Raw sequencing reads (Fastq files) were deposited in the National Center for Biotechnology Information (NCBI) under BioProject accession number PRJNA1308826. Additionally, all code and raw data necessary to replicate the analyses described in this paper have been uploaded to Figshare (DOI: 10.6084/m9.figshare.30227386).
