# Supplementary material for: Eco-evolutionary dynamics sustain a potent yet rare antibiotic gene cluster in Streptomyces
Source: ISME J. 2026 Mar 18;20(1):wrag060. doi: 10.1093/ismejo/wrag060 (PMC13099267; doi:10.1093/ismejo/wrag060)
Supplement: Supplementary_material_wrag060 [file supplementary_material_wrag060.zip › Supplementary Information_S.alb_20260315.pdf]

Supplementary Information

for

Eco-evolutionary dynamics sustain a potent yet rare antibiotic gene cluster in *Streptomyces*

By

Wang et al.

Supplementary material includes:

1) Supplementary methods (PDF), covering bioactivity, metabolite, genomic, and statistical analyses;

2) Figures S1-S14 (PDF), presenting data on genomic analyses, intraspecific competition, antimicrobial activity, and metabolite analysis;

3) Tables S1-S7 (Excel), providing datasets for gene clusters, interaction profiles, kosinostatin BGC analysis, and experimental materials.

Supplemental methods

Agar diffusion assay

To differentiate between interference competition (mediated by diffusible inhibitors) and exploitation competition (driven by nutrient depletion) as potential mechanisms for the growth inhibition observed in the head-to-head competition assays, we performed agar diffusion assays [1]. The sender (bioactive) strains were cultured on GYM agar for 7-20 days. Then, cell-free agar blocks (1 cm^2) were cut from areas immediately adjacent to the culture and placed onto freshly inoculated lawns of the receiver (sensitive) strains. Lawns were periodically checked over 1–5 days for the presence of inhibition zones surrounding the agar blocks. Inhibition was recorded positive when a clear zone of $\geqslant$ 2 mm was observed.

Bioassay-guided fractionation and elucidation of the bioactive compound

To identify the bioactive compound present in the agar blocks of strain FXJ6.189, spore suspensions of FXJ6.189 were streaked onto GYM plates and incubated at 28 °C for 7 days. After fermentation, the agar was extracted twice with an equal volume of ethanol. The combined extract was evaporated to dryness and redissolved in 2 ml methanol. The crude organic extract was then fractionated by analytical HPLC (Shimadzu SPD-M20A diode array detector, detection range 190–800 nm) using an XBridge ODS (octadecylsilyl) column (4.6 × 150 mm, 5 μm particle size) at a flow rate of 1.0 ml/min. Fractions were collected and concentrated for bioactivity assessment. The bioactivity of each fraction was evaluated using a well-diffusion assay as follows: 20 μl of each fraction was loaded into wells made in agar plates previously spread with a spore suspension of strain CR15. After incubation at 28°C for 3 days, the inhibition zones were measured. The bioactive fraction was then subjected to HR-ESI-MS analysis using a Waters Xevo G2 QTOF mass spectrometer equipped with an ACQUITY UPLC BEH C18 column (2.1 mm × 50 mm, 1.7 μm) to identify the compound.

Construction of gene disruption mutants

To confirm that the *ksn* gene cluster is responsible for kosinostatin biosynthesis and to generate a mutant strain deficient in its production, the key biosynthetic gene *ksnC3* was

selected for inactivation based on a previous study [2]. The FXJ6.189Δ*ksnC3* mutant was constructed as follows. Upstream and downstream regions of approximately 2 kb flanking *ksnC3* were amplified from the genomic DNA of strain FXJ6.189 using primer pairs ksnC3-L-For/ksnC3-L-Rev and ksnC3-R-For/ksnC3-R-Rev (Table S6). The kanamycin resistance gene *neo* was amplified from a recombination plasmid pUC119::*neo* using primers neo-For/neo-Rev (Table S6). These PCR products were purified and assembled via Gibson assembly into the *Eco*RV-digested plasmid pKC1139 to yield pKC1139-ksnC3DM. The recombinant plasmid was then introduced into *E. coli* ET12567/pUZ8002 and subsequently transferred into strain FXJ6.189 through conjugation. Spores of exconjugants were harvested and spread on MS agar [3] supplemented with kanamycin. After incubation at 39°C for 5 days, colonies exhibiting apramycin sensitivity and kanamycin resistance were picked out. The colonies were further verified as *ksnC3* disruption mutants (FXJ6.189Δ*ksnC3*) by PCR using primers ksnC3-C-For/ksnC3-C-Rev, followed by sequencing of the resulting amplicons. Independent mutant clones were selected for subsequent analysis of kosinostatin production and antimicrobial activity.

Statistical analysis of BGC distribution drivers

To analyze the drivers of BGC distribution, we constructed three pairwise distance matrices: 1) a BGC distance matrix based on the presence/absence profile of low-frequency BGCs, calculated as Jaccard dissimilarity using the vegdist function in the R package vegan [4]; 2) a geographic distance matrix computed from the latitude and longitude of strain collection sites (where available, see Table S7) using the geosphere [5] package in R; and 3) a phylogenetic distance matrix derived from the concatenated core-gene phylogeny using the ape package [6] in R. The relationships between the distribution of low-frequency BGCs and geographic or phylogenetic distance were assessed using a Mantel test (9,999 permutations) implemented in the vegan package.

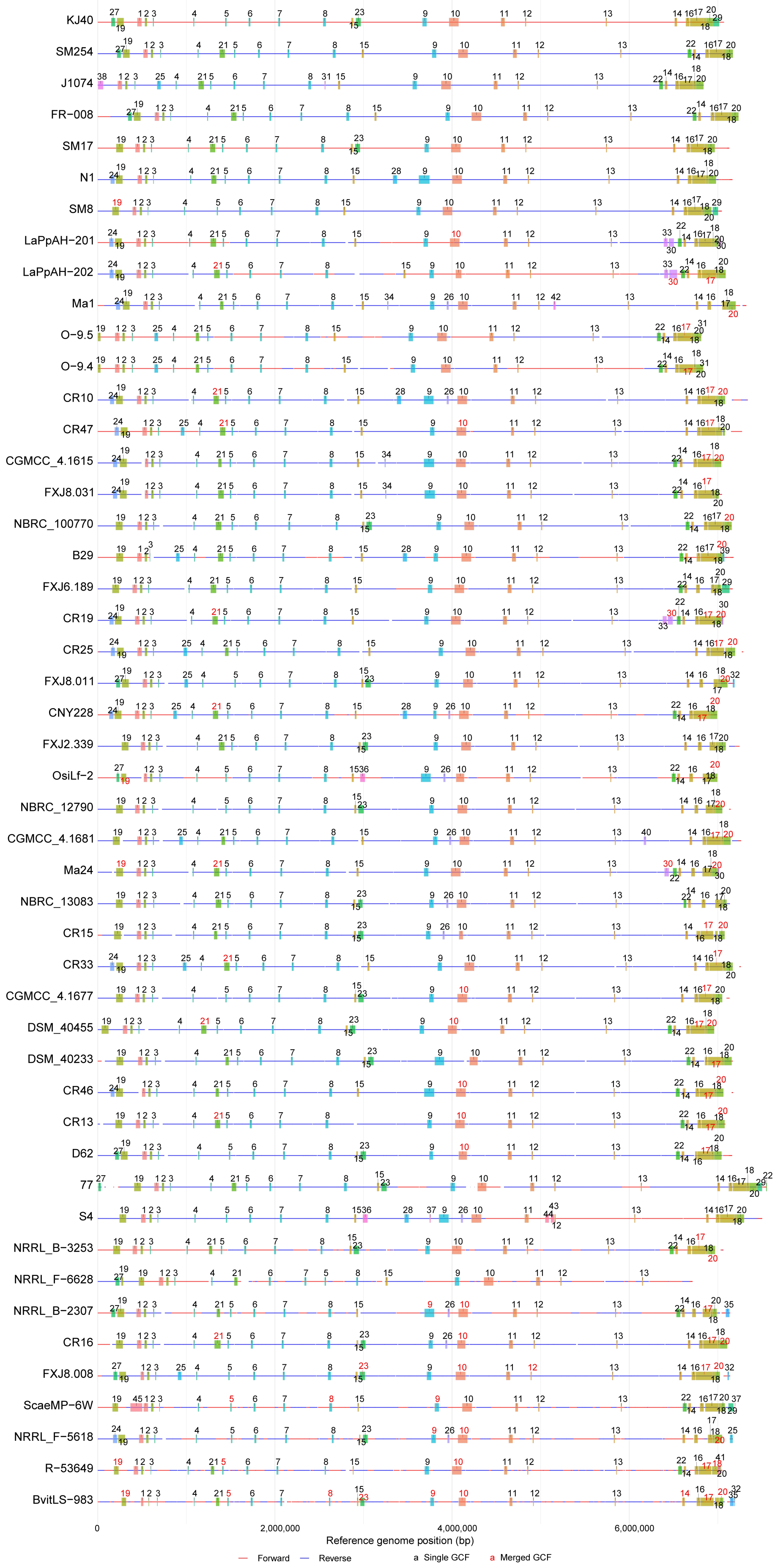
KJ40
SM254
J1074
FR−008
SM17
N1
SM8
LaPpAH−201
LaPpAH−202
Ma1
O−9.5
O−9.4
CR10
CR47
CGMCC_4.1615
FXJ8.031
NBRC_100770
B29
FXJ6.189
CR19
CR25
FXJ8.011
CNY228
FXJ2.339
OsiLf−2
NBRC_12790
CGMCC_4.1681
Ma24
NBRC_13083
CR15
CR33
CGMCC_4.1677
DSM_40455
DSM_40233
CR46
CR13
D62
77
S4
NRRL_B−3253
NRRL_F−6628
NRRL_B−2307
CR16
FXJ8.008
ScaeMP−6W
NRRL_F−5618
R−53649
BvitLS−983
0
2,000,000
4,000,000
6,000,000
Reference genome position (bp)
Forward
Reverse
a Single GCF
a Merged GCF

70

Fig. S1 Genomic distribution of gene clusters in *S. albidoflavus* strains.

Each horizontal line represents a strain genome aligned to reference *S. albidoflavus* KJ40 (red: forward; blue: reverse). Colored rectangles show the positions and lengths of Gene Cluster Families (GCFs), with numeric identifiers. Red labels indicate GCFs that were merged from fragmented BGCs in incomplete assemblies. The x-axis shows reference coordinates (bp); the y-axis shows strains ordered from top to bottom by increasing contig number.

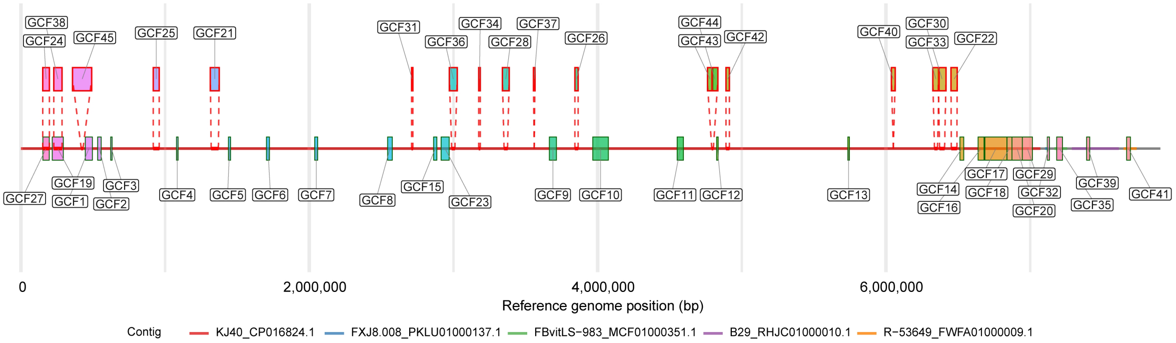

Fig. S2 Mapping of representative GCFs onto a reference-based composite genome. The horizontal line shows a composite genome assembly comprising five segments: the complete *S. albidoflavus* KJ40 reference genome followed by four selected contigs from other strains. Green-bordered rectangles mark the positions of GCFs present within this assembly. Red-bordered rectangles above the line indicate GCFs absent from all segments, connected by dotted lines to putative insertion sites inferred from flanking sequence homology.

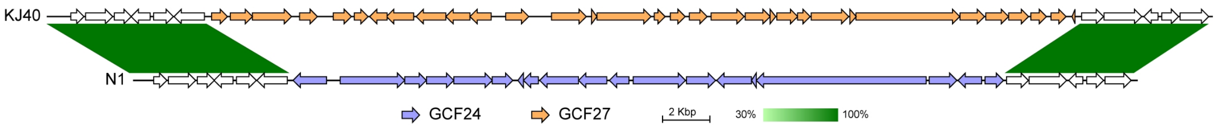

Fig. S3 Genomic evidence for functional replacement of biosynthetic gene clusters. Schematic representation of the genomic locus harboring two gene clusters, GCF24 (encoding an iron-scavenging siderophore) and GCF27 (encoding a copper-scavenging siderophore), in selected *S. albidoflavus* strains. Green bars represent BLASTN identity, with a gradient representing the range from 30% to 100%. The clusters occupy the same genomic position in different strains, indicating a case of functional replacement where distinct but functionally analogous metal-transport gene clusters have been exchanged at a conserved chromosomal site.

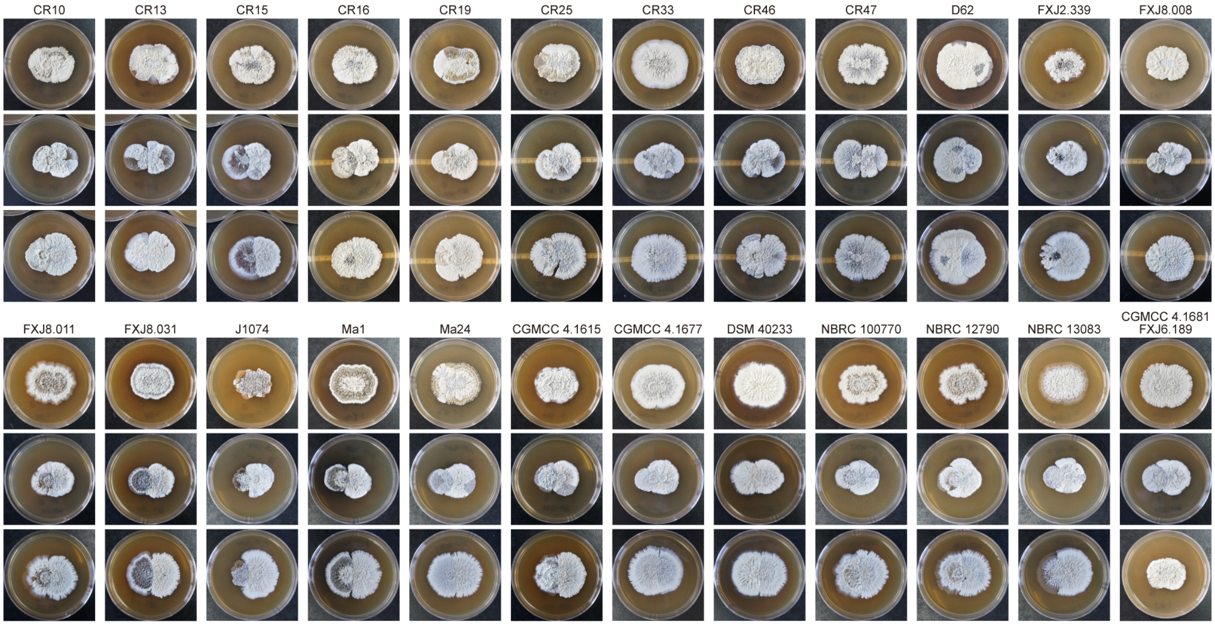

Fig. S4 Pairwise head-to-head competition assays between *S. albidoflavus* strains. Two strains were spotted 0.5 cm apart on the plates and incubated at 28°C for 20 days to evaluate colony size variation. Control plates were inoculated with the same strain spotted against itself. The figure shows outcomes of interactions involving strains FXJ6.189 and CGMCC 4.1681 against other *S. albidoflavus* strains. For each set of three rows, the top row shows self-interaction of a tester strain (control); the middle row shows interaction between the tester strain (left) and FXJ6.189 (right); and the bottom row shows interaction between the tester strain (left) and CGMCC 4.1681 (right). The last column in the second set of three rows depicts: top, self-interaction of strain CGMCC 4.1681; middle, interaction between strains CGMCC 4.1681 (left) and FXJ6.189 (right); bottom, self-interaction of strain FXJ6.189. No change in colony size compared to the self-control was considered a neutral interaction, and reduction in colony size was recorded as growth inhibition. All assays were performed in triplicate.

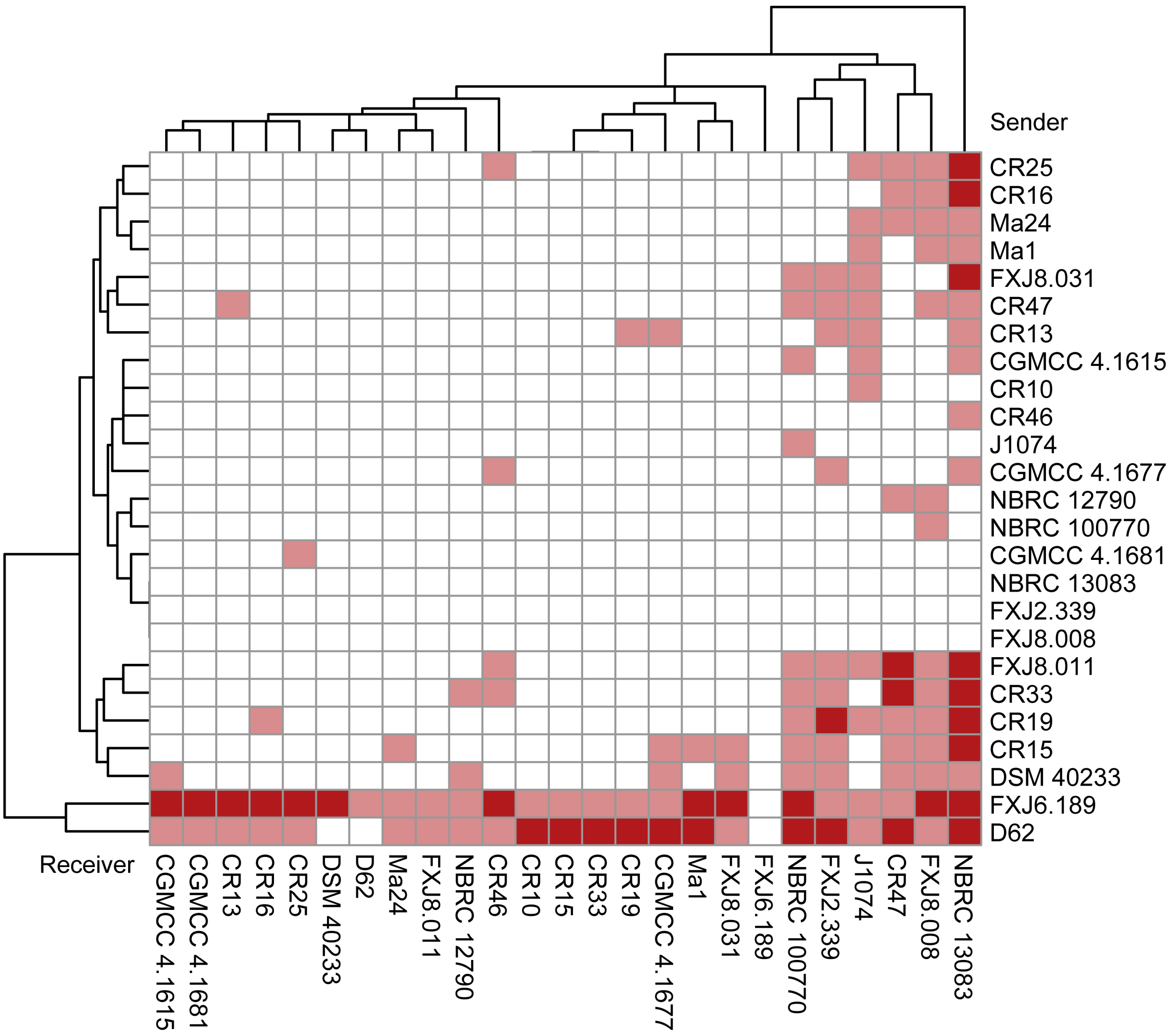

Fig. S5 The intraspecific interaction pattern of *S. albidoflavus*.

Strain numbers of sender and receiver strains are list on the right and bottom of the heatmap, respectively. Hierarchical clustering trees based on interaction profiles are shown above and to the left of the heatmap. Cells colors represent interaction types: neutral (white), weak inhibition (pink), and strong inhibition (red).

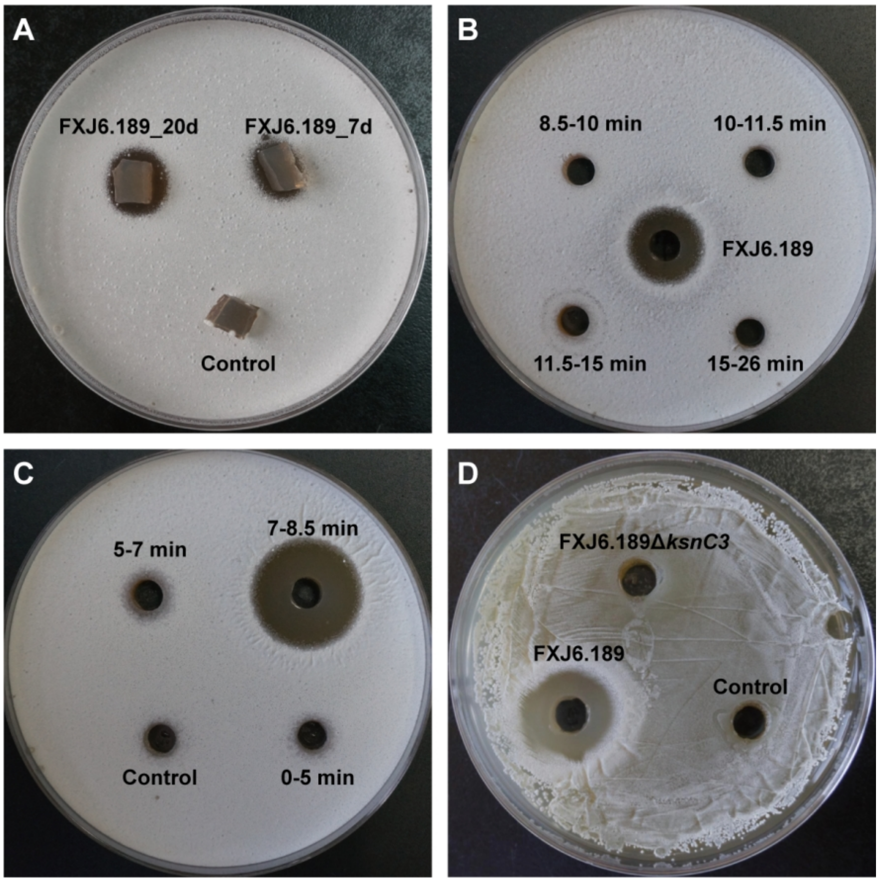

Fig. S6 Antimicrobial activity tests.

(A) Agar diffusion assay. Inhibition zones surrounding agar blocks from strain FXJ6.189 indicate that inhibitory activity observed in the head-to-head competition assays is mediated by diffusible metabolites. (B, C) Bioassay-guided fractionation. Fractions collected form HPLC according to retention time were tested for antimicrobial activity using an agar-well diffusion assay. Bioactive compounds eluted predominantly between 7-8.5 min. (D) Bioactivity comparison between wild-type strain FXJ6.189 and mutant strain FXJ6.189Δ*ksnC3*. Strain CR15 was used as an indicator in these assays.

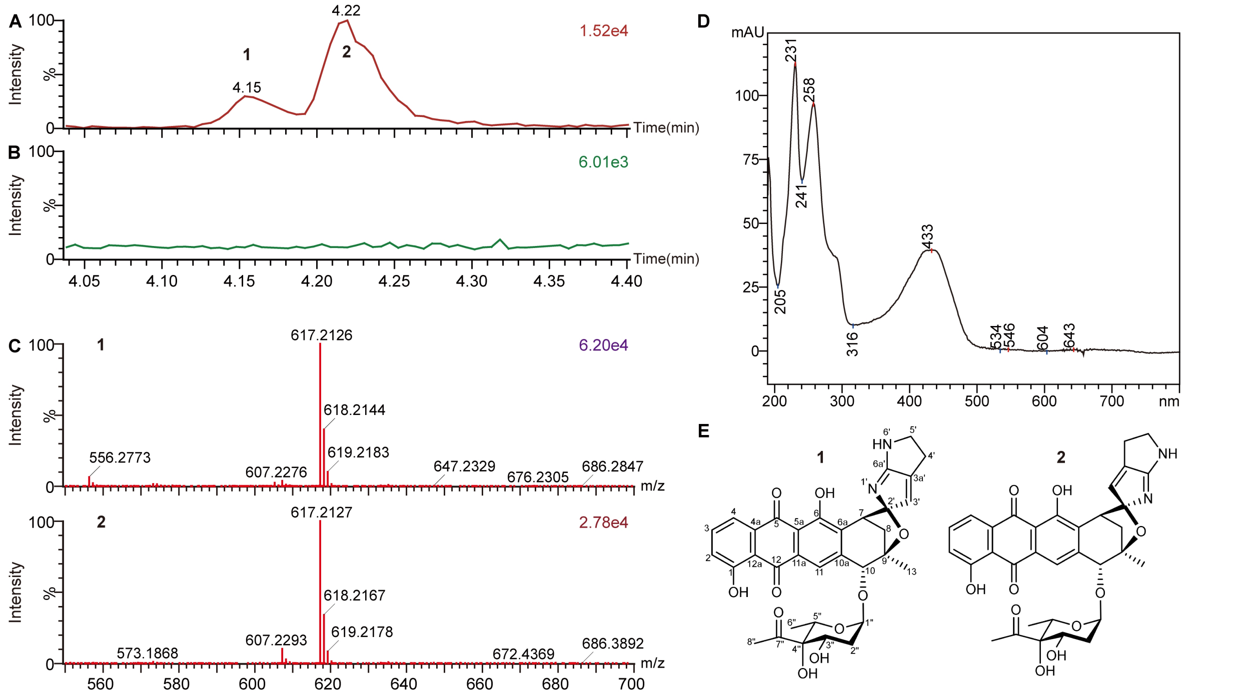

Fig. S7 UHPLC-HRMS analysis of metabolites from wild-type strain FXJ6.189 and mutant strain FXJ6.189Δ*ksnC3*.

(A) UHPLC spectrum of crude extract from wild-type FXJ6.189. (B) UHPLC spectrum of crude extract from mutant FXJ6.189Δ*ksnC3*. (C) HR-ESI-MS spectra ($[M + H]^+$) corresponding to peaks 1 and 2 in (A); (D) UV profiles of peaks 1 and 2 in (A); (E) Chemical structures of kosinostatin (1) and isoquinocycline B (2), reprinted from: Back CR, Stennett HL, Williams SE, et al. A new *Micromonospora* strain with antibiotic activity isolated from the microbiome of a mid-Atlantic deep-sea sponge. Mar Drugs. 2021;19(2):105.

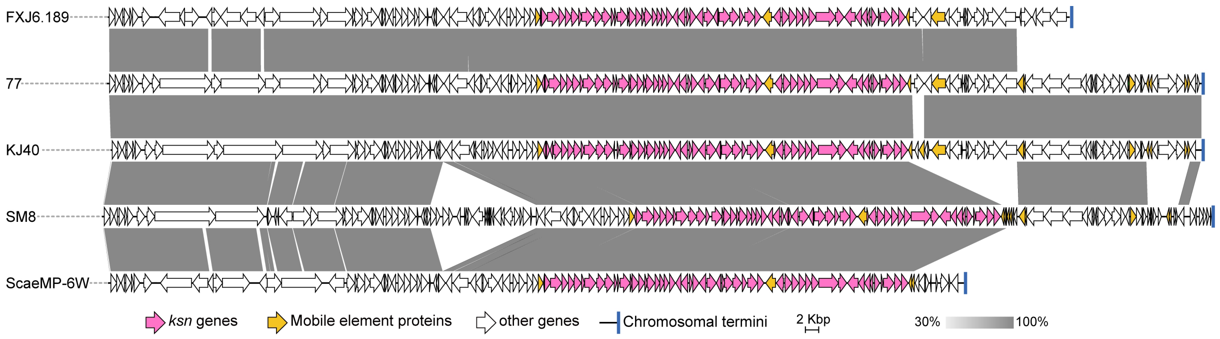

Fig. S8 Synteny analysis of the kosinostatin BGC and flanking sequences in *S. albidoflavus* strains.

Comparative analysis of the genomic context of the *ksn* cluster (shown in pink) shows its consistent location within the plastic terminal region of the chromosome across five *S. albidoflavus* strains. Flanking sequences exhibit an asymmetric conservation pattern: the inner flank (toward the core chromosome) is highly conserved; whereas the outer flank (toward the chromosome end) is variable, featuring indels. This pattern is characteristic of genomic loci acquired through horizontal gene transfer into dynamic, recombination-prone chromosome ends [7].

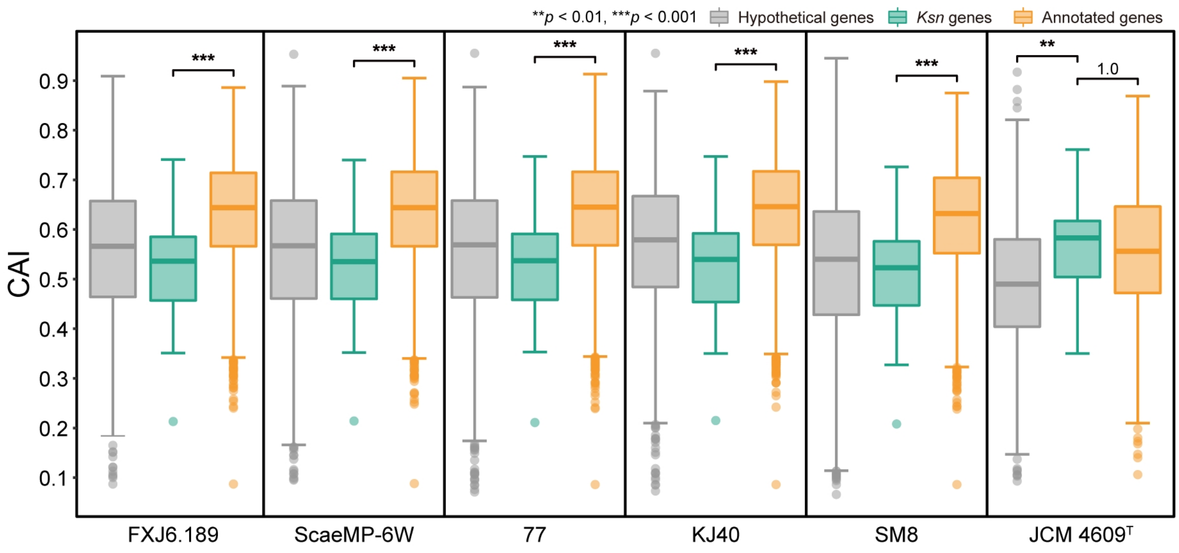

Fig. S9 Codon adaptation index (CAI) analysis of *ksn*-harboring *S. albidoflavus* strains and *S. spiroverticillatus* JCM 4609^{T}.

S. spiroverticillatus JCM 4609^{T} has a kosinostatin BGC highly similar to that of *S. albidoflavus* (Fig. S6). CAI values were compared among three gene categories in each genome: genes within kosinostatin BGCs, annotated genes, and hypothetical genes. The box shows the upper and lower quartiles, the internal line represents the median, and the whiskers extend to 1.5 times the interquartile range. Outliers are plotted as individual points. *P*-values for pairwise comparisons were assessed using one-way ANOVA with Tukey's HSD test.

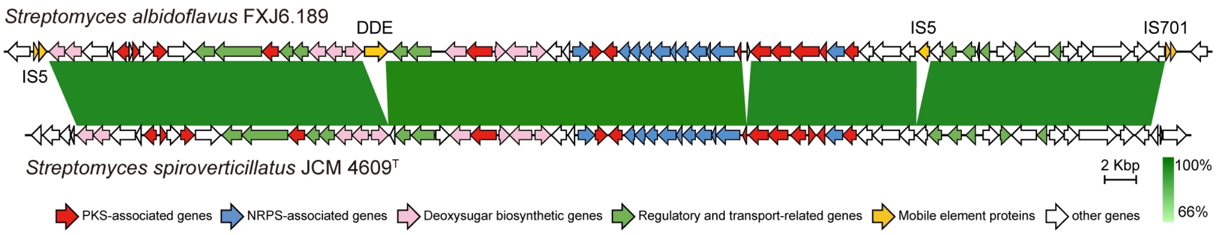

Fig. S10 Comparison of the kosinostatin BGC between *S. albidoflavus* FXJ6.189 and *S. spiroverticillatus* JCM 4609^T. Regions of significant sequence similarity, as determined by BLASTN analysis, are indicated by green shading.

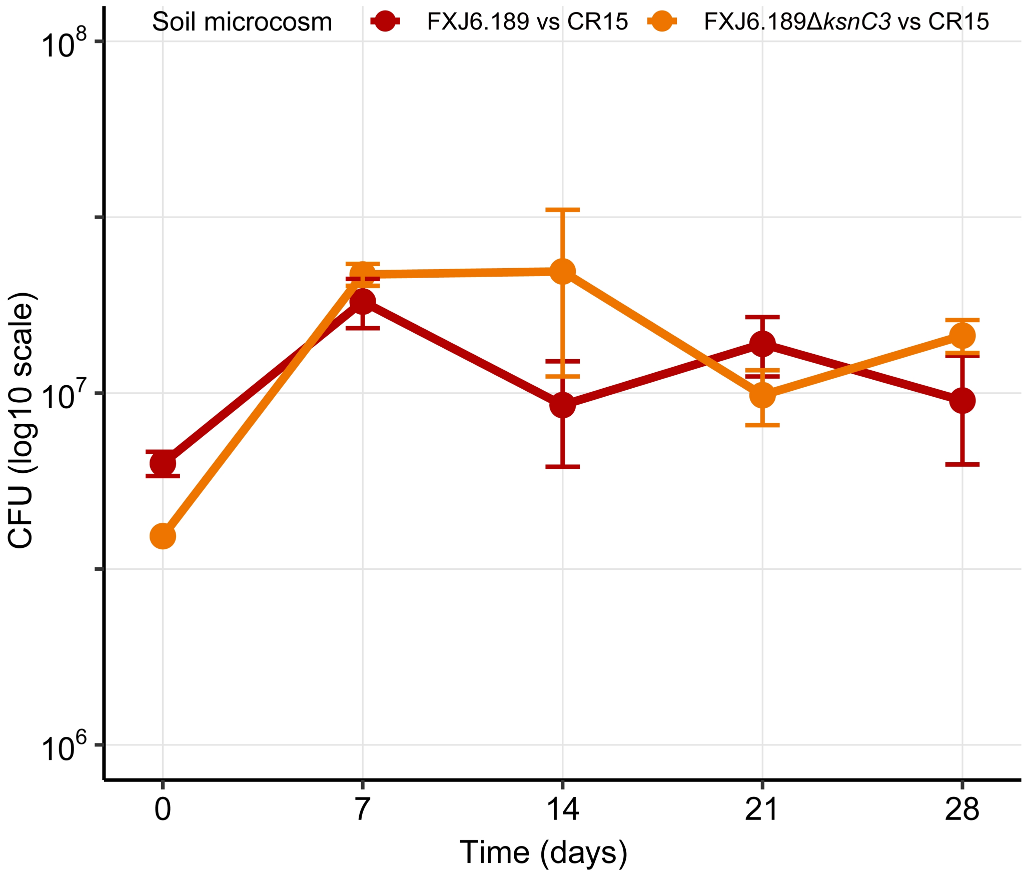

Fig. S11 Population growth dynamics of strains in soil microcosms.

The line graph depicts changes in colony-forming unit (CFU) counts over time for the strains in the soil microcosm experiment. CFUs were quantified via dilution plating of soil suspensions. All populations showed a 3- to 8-fold increase in CFUs after the incubation period compared to the initial inoculation density. This increase confirms successful spore germination and population growth of strains under the experimental soil conditions.

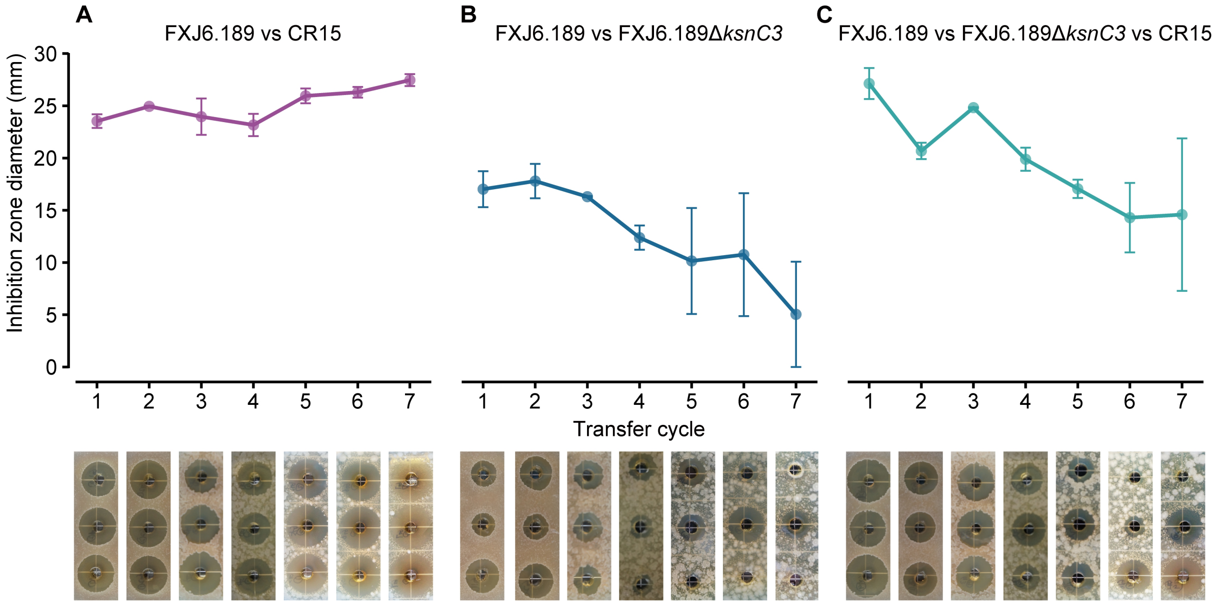

Fig. S12 Dynamics of kosinostatin production in liquid microcosms.

(A–C) Inhibition zone diameters (mean ± SEM, *n*=3) produced by strain FXJ6.189 across serial transfers under the co-culture conditions specified in Figs. 4A and 5AB. Bioactivity was assessed using a well-diffusion assay against *Bacillus subtilis*. Representative assay plates corresponding to each transfer cycle are shown below the horizontal axis, in triplicate per time point. Data for the eighth transfer were not collected as the competitive dynamics had resolved.

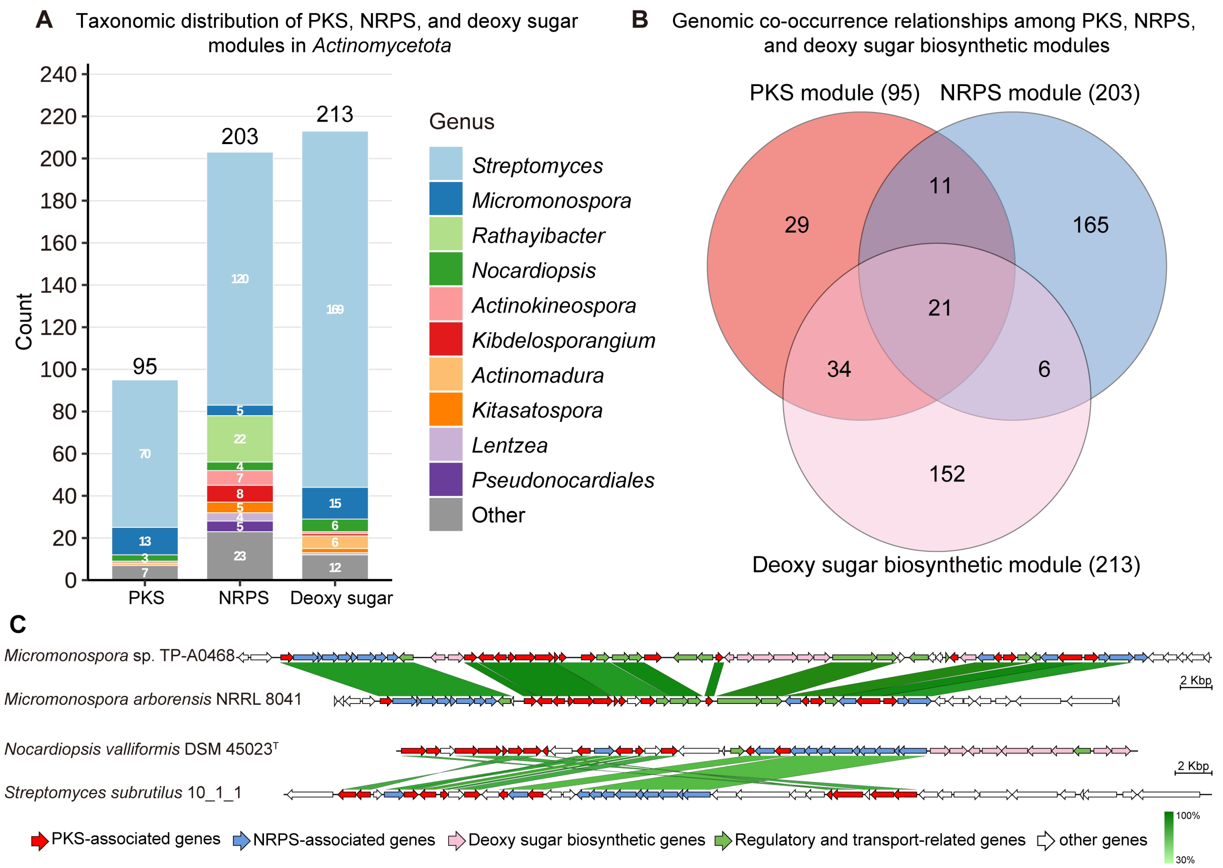

Fig. S13 Distribution of PKS, NRPS, and deoxy sugar biosynthesis module homologs of the kosinostatin BGC across the phylum *Actinomycetota*.

(A) Genus-level distribution of module homologs in *Actinomycetota*. The bar plot shows the number of genera in which homologs of individual modules were detected. (B) Genomic co-occurrence of the three core modules. The Venn diagram shows their distribution across genomes encoding module homologs, revealing that complete co-occurrence is rare. It should be noted that the central intersection includes three more genomes than those containing the intact kosinostatin BGC. Manual inspection confirmed that in these three cases, the homologous modules are scattered at distant genomic loci rather than assembled into a contiguous BGC. (C) Representative partial BGCs. Genomic alignment of two BGCs that contain only subsets of the canonical modules, compared to the intact cluster (top). Both lack the deoxy sugar subcluster, a critical module for kosinostatin biosynthesis. Green bars represent BLASTN identity, with a gradient representing the range from 30% to 100%.

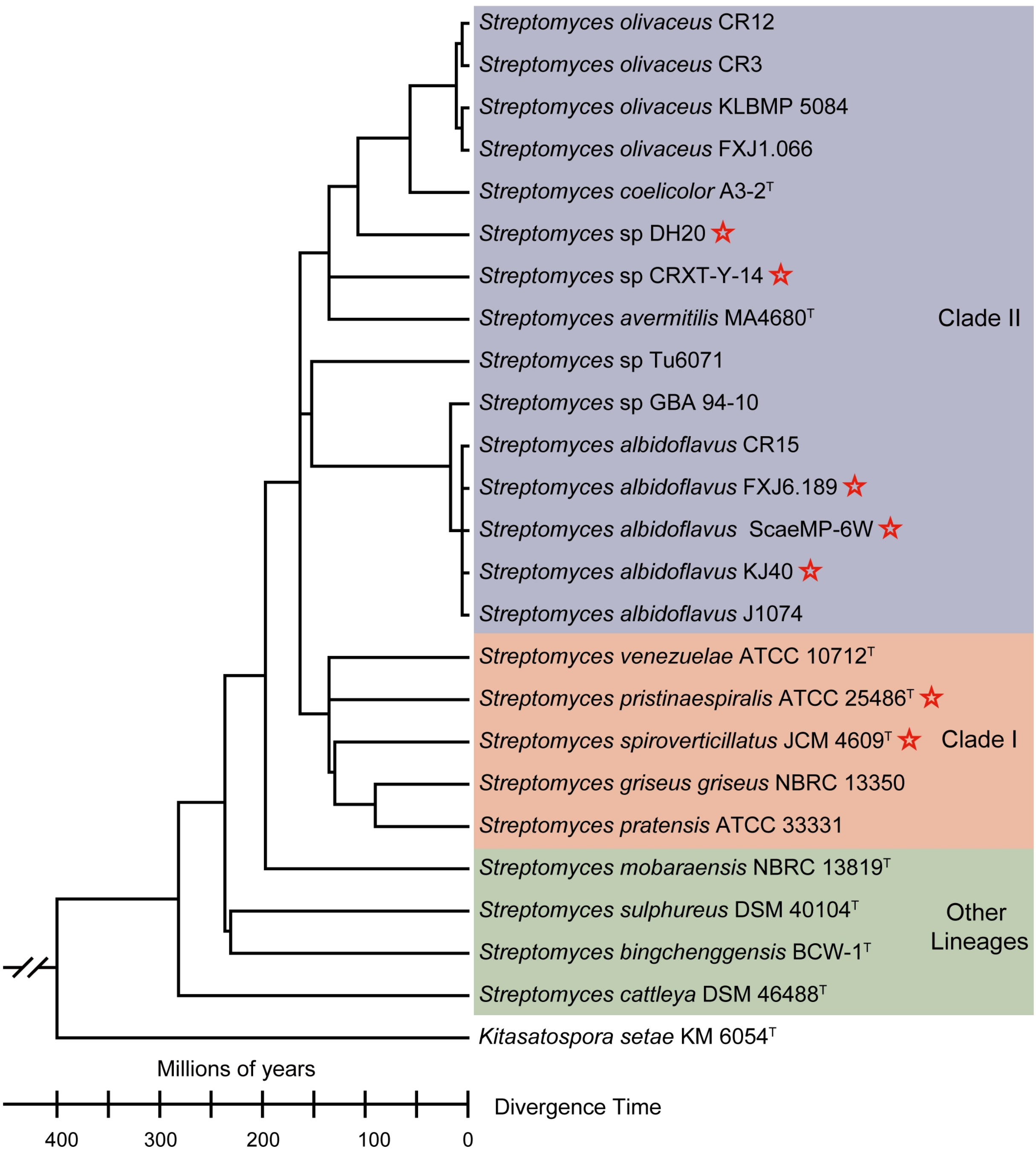

Fig. S14 Molecular clock phylogeny of *Streptomyces*.

The tree was reconstructed using 94 universally conserved bacterial core proteins (GenProp0799). The two major monophyletic clades of *Streptomyces* are marked as Clade I and Clade II as previously reported by McDonald and Currie [8]. Strains harboring kosinostatin BGCs are marked with red stars. Branch lengths correspond to RelTime-estimated divergence times. All nodes received 100% bootstrap support.

References for Supplementary materials

1. Patin NV, Duncan KR, Dorrestein PC *et al.* Competitive strategies differentiate closely related species of marine actinobacteria. *ISME J* 2016;**10**:478-90. https://doi.org/10.1038/ismej.2015.128
2. Ma H-M, Zhou Q, Tang Y-M *et al.* Unconventional origin and hybrid system for construction of pyrrolopyrrole moiety in kosinostatin biosynthesis. *Chem Biol* 2013;**20**:796-805. https://doi.org/10.1016/j.chembiol.2013.04.013
3. Kieser T, Bibb MJ, Chater KF *et al. Practical Streptomyces Genetics: A Laboratory Manual*: John Innes Foundation, 2000.
4. Jari O, Gavin LS, Blanchet FG *et al.* vegan: community ecology package. R package version 2.6-4. https://CRAN.R-project.org/package=vegan (2022).
5. Robert JH. geosphere: Spherical Trigonometry. R package version 1.5-18. https://CRAN.R-project.org/package=geosphere (2022).
6. Emmanuel P, Klaus S. ape 5.0: an environment for modern phylogenetics and evolutionary analyses in R. *Bioinformatics* 2019;**35**:526-28. https://doi.org/10.1093/bioinformatics/bty633
7. Choulet F, Aigle B, Gallois A *et al.* Evolution of the terminal regions of the *Streptomyces* linear chromosome. *Mol Biol Evol* 2006;**23**:2361-69. https://doi.org/10.1093/molbev/msl108
8. McDonald BR, Currie CR. Lateral gene transfer dynamics in the ancient bacterial genus *Streptomyces*. *mBio* 2017;**8**:e00644-17. https://doi.org/10.1128/mBio.00644-17
